# Brain Tumor Class Detection in Flair/T2 Modality MRI Slices Using Elephant-Herd Algorithm Optimized Features

**DOI:** 10.3390/diagnostics13111832

**Published:** 2023-05-23

**Authors:** Venkatesan Rajinikanth, P. M. Durai Raj Vincent, C. N. Gnanaprakasam, Kathiravan Srinivasan, Chuan-Yu Chang

**Affiliations:** 1 Department of Computer Science and Engineering, Division of Research and Innovation, Saveetha School of Engineering, Saveetha Institute of Medical and Technical Sciences, Chennai 602105, India; v.rajinikanth@ieee.org; 2 School of Information Technology and Engineering, Vellore Institute of Technology, Vellore 632014, India; pmvincent@vit.ac.in; 3 Department of Electronics and Instrumentation Engineering, St. Joseph’s College of Engineering, OMR, Chennai 600119, India; gnanacn@gmail.com; 4 School of Computer Science and Engineering, Vellore Institute of Technology, Vellore 632014, India; kathiravan.srinivasan@vit.ac.in; 5 Department of Computer Science and Information Engineering, National Yunlin University of Science and Technology, Yunlin 64002, Taiwan; 6 Service Systems Technology Center, Industrial Technology Research Institute, Hsinchu 310401, Taiwan

**Keywords:** brain-tumor classification, deep learning, feature optimization, MRI slice, classification

## Abstract

Several advances in computing facilities were made due to the advancement of science and technology, including the implementation of automation in multi-specialty hospitals. This research aims to develop an efficient deep-learning-based brain-tumor (BT) detection scheme to detect the tumor in FLAIR- and T2-modality magnetic-resonance-imaging (MRI) slices. MRI slices of the axial-plane brain are used to test and verify the scheme. The reliability of the developed scheme is also verified through clinically collected MRI slices. In the proposed scheme, the following stages are involved: (i) pre-processing the raw MRI image, (ii) deep-feature extraction using pretrained schemes, (iii) watershed-algorithm-based BT segmentation and mining the shape features, (iv) feature optimization using the elephant-herding algorithm (EHA), and (v) binary classification and verification using three-fold cross-validation. Using (a) individual features, (b) dual deep features, and (c) integrated features, the BT-classification task is accomplished in this study. Each experiment is conducted separately on the chosen BRATS and TCIA benchmark MRI slices. This research indicates that the integrated feature-based scheme helps to achieve a classification accuracy of 99.6667% when a support-vector-machine (SVM) classifier is considered. Further, the performance of this scheme is verified using noise-attacked MRI slices, and better classification results are achieved.

## 1. Introduction

The disease rate in mankind is rising progressively globally due to various reasons, and it is causing a heavy diagnostic burden to hospitals. Many recent procedures and aiding schemes have been developed to reduce these burdens to support the doctors who perform initial screening, disease-severity confirmation, treatment planning, and executing and monitoring recovery rate [1]. Furthermore, the development in computing facilities and improvement in artificial-intelligence (AI) schemes also helps reduce the diagnostic burden by implementing several machine-learning (ML) and deep-learning (DL) procedures to support various levels of disease diagnostic processes.

The 2020 report by the Global Cancer Observatory (GCO) confirmed that the incidence and death rate due to cancer is gradually increasing globally. Therefore, early detection is essential to completely cure the disease using the chosen medication procedures. Brain tumor is a medical emergency, and an early and accurate diagnosis is indispensable to execute promising treatment. In the GCO report, cancer associated with the brain was listed in the 19th position based on the incidence (308,102 cases) and in the 12th position based on the confirmed death rate (251,329 cases). Because of its severity, several measures are suggested by researchers to detect brain cancer using a chosen approach [2]. Therefore, accurate detection of cancer and its category is necessary and vital when treating patients.

There are severe physical and psychological issues associated with brain tumors (BTs) in humans. In order to determine how to treat them, an accurate diagnosis is necessary. As a consequence, the World Health Organization (WHO) issued guidelines to assist in the identification of brain tumors (BTs) and the class of each of these tumors in order to plan and implement the necessary treatment [3]. It is common practice in hospitals to perform medical-image verification procedures when it comes to the diagnosis of BTs, and radiological approaches, such as computed tomography (CT) and magnetic-resonance imaging (MRI), are standard procedures to be followed when a diagnosis of BT is determined. There are various techniques that can be used to carry out MRI; however, the FLAIR and T2 modalities tend to be the most commonly used ones due to their superior visibility compared to the T1, T1-contrast (T1C), and diffused-weight (DW) techniques, and hence are widely adopted due to their multi-modality nature [4].

The clinical-level exposure of the BT involves segmenting the affected section to confirm the harshness and position of the tumor in the brain. Further, computer algorithms are also widely employed in modern hospitals to examine the radiological images to detect BTs with improved accuracy. Computerized procedures, combined with recent AI schemes such as ML and DL techniques, are commonly employed in (i) classification and (ii) segmentation tasks, which helps to lessen the investigative time noticeably. The work of Rajinikanth et al. [5] on BT detection based on MRI slices confirmed that computer algorithms act as an aiding tool for the radiologist and help to provide the initial level-assessment report. The neurologist confirms this report and finally decides on the treatment to be executed [6].

Recent works on BT detection confirm that DL approaches help to attain improved accuracy compared with ML schemes [7,8]. Hence, researchers have developed several DL schemes to examine benchmark and clinical MRI slices with the tumor [9,10]. Further, earlier works also confirmed that the outcome of the pretrained DL structure can be upgraded by employing optimization-algorithm-based feature selection [11], an ensemble of feature approach [12], fused deep features [13], and serially integrated DL and ML features [14]. The goal of any computer algorithm is to achieve an enhanced BT recognition irrespective of the MRI modality and its quality. The former research on BT detection can be accessed in [15,16].

This research develops a BT-detection framework using serially integrated ML and DL features to achieve better accuracy during FLAIR- and T2-modality image analysis. This framework involves the subsequent stages: (i) data collection, 3D to 2D transformation, and pre-processing; (ii) deep-feature extraction using a pretrained scheme; (iii) feature mining of the shape of the tumor; (iv) feature selection using the elephant-herding algorithm (EHA); and (v) binary classification and verification using three-fold cross-validation. This work investigates the following DL methods: VGG16, VGG19, ResNet50, ResNet101, and DenseNet101. Further, watershed segmentation (WS) is also considered to segment the tumor region to obtain the necessary ML features, such as gray-level co-occurrence matrices (GLCMs) and Hu moments. The serial integration of DL and ML (DL + ML) features is considered to achieve better BT detection.

Using EHA-based optimization, this paper reduces the DL features in order to avoid the issue of overfitting. As a first step towards verifying the significance of the proposed scheme in this study, well-known benchmark BT datasets, such as BRATS2015 [17,18] and TCIA [19,20], are utilized. The results of this study indicate that the proposed work is significant for analyzing FLAIR/T2 MRI slices. It is also confirmed that a clinical significance can be derived from taking into consideration the MRI slices that are associated with noise (Gaussian noise) as part of the proposed technique. According to the experimental result of this study, with the serially fused features of the considered axial-plane MRI slices, the developed classification scheme contributes to a classification accuracy greater than 99% in cases of the considered axial-plane MRI slices.

The contributions of this research work are discussed below:

- Development of a unique procedure to examine FLAIR- and T2-modality MRI slice with/without the skull region;
- Integrated DL and ML features to achieve better BT-detection performance;
- EHA-based feature optimization to obtain better results without the overfitting issue;
- Verifying the performance of the proposed scheme using a clinical MRI dataset of the T2-modality.

Section 2 presents the previous studies on BT classification, Section 3 illustrates the methodology, and Section 4 and Section 5 present the results and conclusion of this study, respectively.

## 2. Literature Review

The development of a computerized algorithm for automatically detecting BT in a chosen MRI slice has gained attention among researchers [21,22,23]. Hence, several automatic procedures have been proposed and implemented to assess various modalities of MRI slices. In recent procedures, binary classifiers were used to classify MRI slices using customized/pre-trained deep-learning methods, resulting in more accurate results. In addition, several procedures were implemented in earlier studies to improve BT-detection accuracy, and Table 1 summarizes a few chosen approaches.

The above table summarizes a few earlier works that implemented the BRATS and TCIA databases, and the maximum accuracy presented in these works was 99.29%. Hence, this works implemented a methodology to improve BT detection to achieve an accuracy of >99.29%.

## 3. Methodology

When brain MRI is taken into account, the DL-based BT-detection procedure can result in a higher degree of accuracy. Thus, the purpose of this research was to develop an appropriate BT-detection procedure that can be used to analyze MRI slices of the FLAIR and T2 modalities. Moreover, the proposed scheme was tested on MRI slices corrupted with noise in order to confirm its ability to detect BTs in the presence of adversarial attacks. Thus, even if the MRI is associated with noise, the proposed deep-learning scheme would be able to detect BT efficiently.

### 3.1. Disease-Detection Scheme

**Figure 1:**
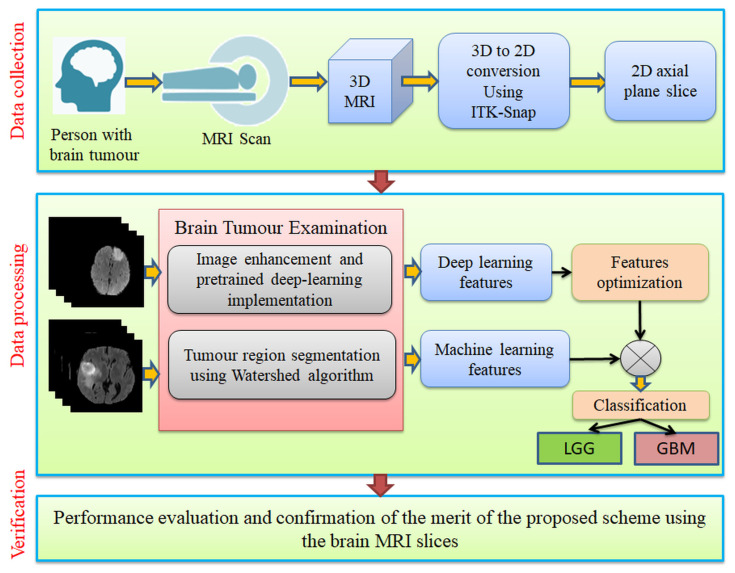
Structure of the proposed scheme to examine BT in MRI slices of FLAIR/T2 modalities with and without the skull.

As depicted in Figure 1, this study collected the necessary MRI data from the benchmark datasets (BRATS2015 and TCIA). As these images were in 3D format, the ITK-Snap tool [41] was used to obtain 2D slices (axial plane) from them, and these slices were used to confirm the merit of the developed algorithm. This framework utilizes deep-learning features to classify MRI slices and improve detection performance. Following the extraction of the tumor section using WS, the DL features are serially combined with the ML features. In addition, this technique was optimized to minimize overfitting results by using EHA-based feature selection. Finally, to verify the eminence of the developed system on MRI slices with or without a skull section, binary classification with a three-fold cross-validation methodology was implemented, followed by evaluating the achieved performance metric.

In order to achieve superior detection accuracy when it comes to BT when both FLAIR- and T2-modality MRI are considered, the technique executes a novel procedure that combines the features of DL and ML to achieve a better detection accuracy by integrating the features of the two techniques. Based on the benchmark images, this scheme was tested and verified, and it was confirmed from the proposed outcomes that this scheme will help in achieving better accuracy in benchmark images. As a result, it was confirmed that this scheme is better and can be considered for use in order to examine clinical MRI images with the skull section as well.

### 3.2. MRI Dataset

The availability of clinical-grade brain-MRI slices for research purposes is minimal, as discussed in [1], and accurate clinical MRI images are protected due to ethical issues. Most of these images are available for clinical study instead of academic research. Hence, most of the research considered the benchmark-image datasets (authentic clinical and synthetic images) available for academic research. This work considered the Brain Tumor Segmentation (challenge database (BRATS2015)) and The Cancer Imaging Archive (TCIA) datasets for the examination. From BRATS, skull-stripped FLAIR-MRI slices of low-/high-grade glioma (LGG/HGG) were considered for examination, and earlier works on these images can be found in [17,18]. The TCIA database consists of LGG- [19] and glioblastoma (GBM)-class MRI [20], and earlier works on these databases can be accessed in [25,26,27,28].

**Figure 2:**
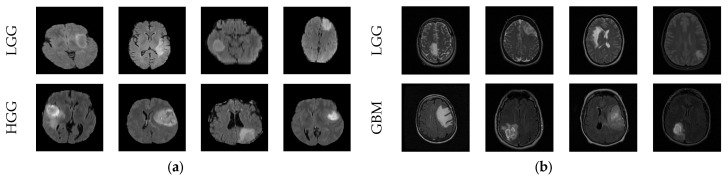
Sample test images of BRATS and TCIA. (**a**) FLAIR-modality MRI slices collected from BRATS, (**b**) T2-modality MRI slices collected from TCIA.

BRATS and TCIA provided a 3D picture, from which 2D slices were obtained using ITK-Snap, and these slices were then considered to authenticate the performance of the planned system. This work considered 1500 MRI slices (dimension of pixels) from each class for the examination. Every image was resized to the required size and enhanced using the CLAHE technique to advance prominence of the tumor section. The sample test-image set is shown in Figure 2, and the considered image value is presented in Table 2. Figure 2a,b show the FLAIR- and T2-modality slices. In Table 2, Class 1 represents LGG and Class 2 represents HGG/GBM. The earlier DL-based classification of these datasets can be found in [1,4,5].

**Figure 3:**
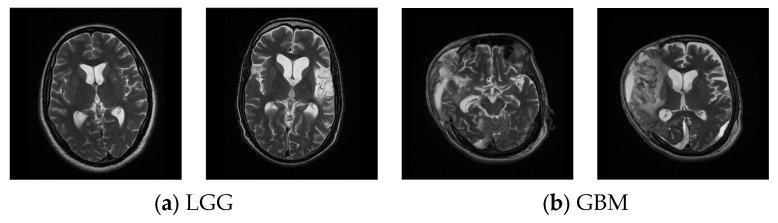
Clinically collected T2-modality MRI slices. (**a**) LGG, (**b**) GBM.

Along with the benchmark-image datasets, this research also considered clinically collected T2-modality MRI slices from real patients, as shown in Figure 3. Figure 3a,b present the LGG and GBM images from the clinical dataset [42,43].

### 3.3. Feature Extraction

For this study, DL and ML features were considered for the examination, as the performance of ML and DL schemes was primarily determined by the information extracted from the chosen test images.

#### 3.3.1. Deep Features

The literature on BT detection in MRI with the DL scheme confirms that DL procedures help achieve better detection than other available image-examination schemes. Hence, DL-based BT-detection procedures have been widely adopted by researchers [26,27,28,29,30,31,32]. The following initial values were assigned for the chosen DL schemes: learning rate = 1 × 10^−5^, Adam optimization, ReLu activation, total iteration = 1000, total epochs = 150, and classification with SoftMax unit using three-fold cross-validation. The proposed work considered the mined features to classify the chosen MRI slices. Every DL scheme was allowed to produce a one-dimensional feature vector of size, and it was then considered for the disease-detection task as in Equations (1) and (2).

(1) $${\mathrm{DLfeature}}_{\mathrm{VGG}16\left(1\times 1\times 1000\right)}=\mathrm{VGG}16_{\left(1,1\right)},\mathrm{VGG}16_{\left(1,2\right)},\ldots ,\mathrm{VGG}16_{\left(1,1000\right)}$$

(2) $${\mathrm{DLfeature}}_{\mathrm{DenseNet}\left(1\times 1\times 1000\right)}={\mathrm{DenseNet}}_{\left(1,1\right)},{\mathrm{DenseNet}}_{\left(1,2\right)},\ldots ,{\mathrm{DenseNet}}_{\left(1,1000\right)}$$

#### 3.3.2. Tumor Features

**Figure 4:**
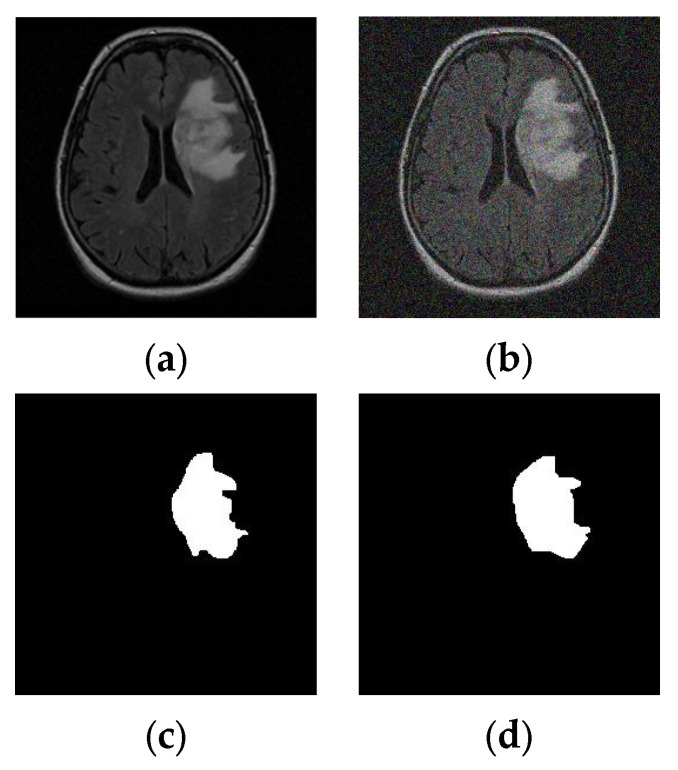
Tumor-section extraction from the chosen MRI slice with the watershed algorithm. (**a**) Original image, (**b**) noisy image, (**c**) tumor from original image, (**d**) tumor from noisy image.

Machine-learning features such as GLCMs and Hu moments were considered in addition to DL features to improve BT detection. The ML and DL features were combined in this work to improve accuracy, and this process is discussed in this subsection. More information about these features can be found in [5,11]. In this work, the watershed-segmentation (WS) procedure was used to mine the MRI slices of the tumor. A WS scheme uses an automatic algorithm to mine the tumor region through edge detection, morphological operations, tumor enhancements, and extractions. The marker dimension primarily determines the WS scheme; in this case, it was assigned a score of 10 [44]. Figure 4 depicts the result of WS for a chosen image from TCIA and Figure 4a,b depict the original image and a noise-corrupted image. Figure 4c,d show the segmented tumor, which helped to achieve the necessary tumor features, such as GLCMs and Hu moments [4,5]. The binary tumor section was considered to obtain 25 GLCM features and three Hu features. The merit of the WS was confirmed with delicate and noisy images, and the proposed segmentation approach worked well on fine and noise-corrupted images.

The proposed WS effectively extracted the tumor in LGG-, HGG-, and GBM-class MRI slices. The extracted BT was in the form of the binary, and it effectively provided the GLCMs and Hu moments. These features were considered to improve the BT-detection accuracy by integrating it with the DL features. The ML features considered in this study can be found in Equations (3) and (4):

(3) $${\mathrm{GLCM}}_{\left(1\times 1\times 25\right)}={\mathrm{GLCM}}_{\left(1,1\right)},{\mathrm{GLCM}}_{\left(1,2\right)},\ldots ,{\mathrm{GLCM}}_{\left(1,25\right)}$$

(4) $${\mathrm{Hu}}_{\left(1\times 1\times 3\right)}={\mathrm{Hu}}_{\left(1,1\right)},{\mathrm{Hu}}_{\left(1,2\right)},{\mathrm{Hu}}_{\left(1,3\right)}$$

#### 3.3.3. Feature Optimization

When a large amount of data information is considered in a disease-detection task, ML and DL may provide an over-fitting result, which must be avoided to validate the importance of this technique. Because of this, feature-selection/-optimization schemes have been extensively employed in the literature, and the reduced feature has then been considered to verify the performance of the implemented scheme. However, the feature-reduction task can be implemented using the conventional approach known as Student’s t-test, which involves more computation to achieve a reduced feature vector. Hence, several heuristic algorithms have recently been employed to optimize the features, and in this work, the elephant-herding-algorithm (EHA)-based procedure was implemented for the data selection.

The EHA is a heuristic scheme developed by mimicking the food-foraging behavior of an elephant herd, which is guided by a group leader (matriarch). The essential information for the EHA can be found in [45]. The theory behind the EHA includes the following conditions: Each clan contains a fixed number of members (elephants) in the herd, and the adult male elephants leave the herd and live alone during each generation. An older matriarch commands each clan.

In order for the EHA to work, the following assumptions must be made:

- The herd of elephants in each clan is stable;
- Male elephants are separate from their groups in each generation;
- Herds are led to food and water by older elephants (matriarchs).

**Figure 5:**
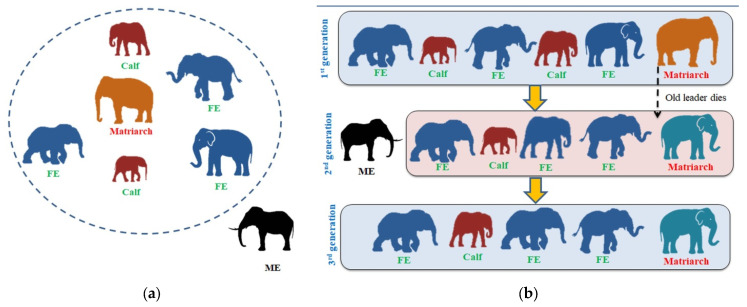
The working procedure of EHA. (**a**) Initial EHA, (**b**) EHA-based optimization.

The clan-updating and -separating process makes up the EHA-optimization search. An overview of the EHA is shown in Figure 5. Figure 5a,b present the initial distribution of elephants in the search space.

Equations (5)–(7) show the mathematical expression of the algorithm:

(5) $$X_{new,ci,j}=X_{ci,j}+À*X_{best,ci}-X_{ci,j})*ℜ$$

where ci denotes the matriarch of the clan, $X_{ci,j}$ is the earlier location, $X_{new,ci,j}$ is the updated location, *j* is the total number of elephants in the clan, and the random value of range $\epsilon \left[0,1\right]$ is denoted as À and ℜ.

New positions are also detected during each operation:

(6) $$X_{new,ci,j}=\beta *X_{center,ci,d}$$

where $X_{center,ci,d}=\frac{1}{n_{ci}}\times \sum_{i=1}^{n_{ci}}X_{ci,j,d}$ and *d* denotes the search dimension.

The performance of the separation process improves based on the number of generations. During this process, the male elephant is discarded and the positions of the other elephants are updated. This process is depicted in Figure 4b, and its mathematical expression can be seen in Equation (7):

(7) $$X_{worst,ci}=X_{min}+\left(X_{max}-X_{min}+1\right)*rand$$

where $X_{min}\;\mathrm{and}\;X_{max}$ are the limits, and rand is a random number.

The optimization process with the EHA can be found in [46], and in this work, it was considered to optimize the DL features. Similar work related to this task can be found in [47]. The initial parameters of the EHA were assigned as follows: number of members = 30, number of generations = 5, dimensions of search = 2, objective value = Cartesian distance, number of iterations $\left(Iter_{max}\right)$ = 1500, and algorithm termination = $Iter_{max}$. The feature optimization helped reduce VGG16 and DenseNet101 features to a lower value, combined serially to improve the features. The EHA-based approach helped reduce the features to a lower level when the FLAIR-modality MRI was used, and this value is presented in Equations (8) and (9). Similarly, the reduced feature for the T2-modality MRI is shown in Equations (10) and (11):

(8) $${\mathrm{DLfeature}}_{\mathrm{VGG}16\left(1\times 1\times 373\right)}=\mathrm{VGG}16_{\left(1,1\right)},\mathrm{VGG}16_{\left(1,2\right)},\ldots ,\mathrm{VGG}16_{\left(1,373\right)}$$

(9) $${\mathrm{DLfeature}}_{\mathrm{DenseNet}\left(1\times 1\times 416\right)}={\mathrm{DenseNet}}_{\left(1,1\right)},{\mathrm{DenseNet}}_{\left(1,2\right)},\ldots ,{\mathrm{DenseNet}}_{\left(1,416\right)}$$

(10) $${\mathrm{DLfeature}}_{\mathrm{VGG}16\left(1\times 1\times 401\right)}=\mathrm{VGG}16_{\left(1,1\right)},\mathrm{VGG}16_{\left(1,2\right)},\ldots ,\mathrm{VGG}16_{\left(1,401\right)}$$

(11) $${\mathrm{DLfeature}}_{\mathrm{DenseNet}\left(1\times 1\times 428\right)}={\mathrm{DenseNet}}_{\left(1,1\right)},{\mathrm{DenseNet}}_{\left(1,2\right)},\ldots ,{\mathrm{DenseNet}}_{\left(1,428\right)}$$

### 3.4. Implementation

**Figure 6:**
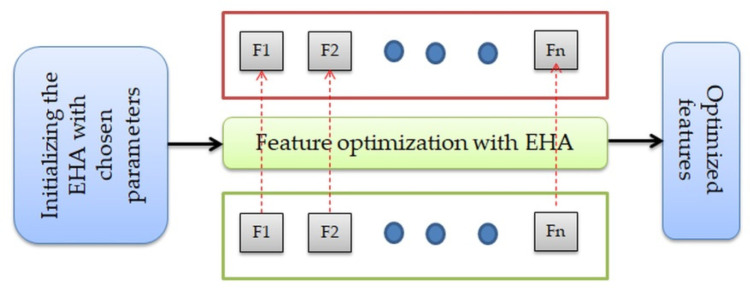
Feature-selection process with the EHA.

The experimental investigation of this study was implemented using a workstation with the following specifications: Intel i7, 16GB RAM, and 4GB VRAM. The procedures, such as tumor segmentation with WS and EHA-based feature optimization, were implemented using MATLAB. Other prime tasks, such as DL-feature mining and classification, were executed using PYTHON. The proposed work was separately tested and verified on the MRI slices of the FLAIR and T2 modalities, and the achieved results were recorded and analyzed. The dual deep features of this study were obtained by serially integrating the VGG16 and DenseNet101 features, and the DL+ML feature was obtained by serially combining the VGG16, GLCMs, and Hu moments. The feature optimization with the proposed EHA is shown in Figure 6. In this process, the optimal features were selected by comparing the tumor features (F1, F2,…, Fn) and considering the Cartesian distance (CD). Finally, the feature with the maximal CD was selected, and other features were discarded.

In this work, the feature dimension considered for the FLAIR modality was as follows: Dual−deep=1×1×789 and DL+ML=1×1×401. Similarly, the T2-modality examination was performed using the following features: Dual deep=1×1×829 and DL+ML=1×1×429. These features were then considered to classify the MRI using binary classification with three-fold cross-validation.

### 3.5. Performance Evaluation

A high-quality approach must be demonstrated before it can be recommended for clinical MRI assessment. The performance of the proposed system was verified by computing true-positive (TP), true-negative (TN), false-positive (FP), and false-negative (FN) metrics. These values were then used to calculate accuracy, precision, sensitivity, and specificity. A binary classifier such as SoftMax, decision tree (DT), random forest (RF), K-nearest neighbor (KNN), and support-vector machine (SVM) was considered and the results were recorded [48,49].

The mathematical expression of these measures can be found in Equations (12)–(16):

(12) $$\mathrm{Accuracy}\;\left(\mathrm{AC}\right)=\frac{\mathrm{TP}+\mathrm{TN}}{\mathrm{TP}+\mathrm{TN}+\mathrm{FP}+\mathrm{FN}}\times 100$$

(13) $$\mathrm{Precision}\;\left(\mathrm{PR}\right)=\frac{\mathrm{TP}}{\mathrm{TP}+\mathrm{FP}}\times 100$$

(14) $$\mathrm{Sensitivity}\;\left(\mathrm{SE}\right)=\frac{\mathrm{TP}}{\mathrm{TP}+\mathrm{FN}}\times 100$$

(15) $$\mathrm{Specificity}\;\left(\mathrm{SP}\right)=\frac{\mathrm{TN}}{\mathrm{TN}+\mathrm{FP}}\times 100$$

(16) $$F1-\mathrm{Score}\;\left(F1S\right)=\frac{2\mathrm{TP}}{2\mathrm{TP}+\mathrm{FP}+\mathrm{FN}}\times 100$$

## 4. Results and Discussion

**Figure 7:**
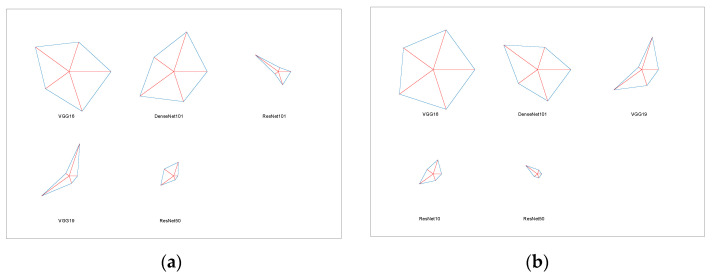
Glyph plot of Table 3 values. (**a**) LGG/HGGs, (**b**) LGG/GBMs.

The experimental results of this study are presented in this section. The proposed scheme was initially implemented in the BRATS2015 database (FLAIR MRI without skull), and the result of the LGG/HGG classification was recorded. First, the classification task was performed using the individual DL features, and Table 3 illustrates the results obtained from this experiment. Based on the classification-accuracy values of the DL schemes, this table indicates that VGG16 and DenseNet101 performed better on the chosen MRI slices than VGG19, ResNet50, and ResNet101. The same experiment was conducted with the TCIA dataset (T2 MRI with skull), and the obtained results during LGG/GBM classification can also be seen in Table 3. In addition, this confirms that VGG16 and DenseNet101 had higher classification accuracy than the other schemes in this study. Figure 7 presents the overall comparison of the performance metrics, with Figure 7a depicting LGG/HGGs and Figure 7b depicting LGG/GBMs.

Following identification of the best DL scheme, the proposed research was repeated using a dual deep feature and a serially integrated DL + ML scheme. Along with SoftMax, other classifiers were also considered during this process, and each approach was noted based on the performance measure achieved. Three-fold cross-validation was performed during this task, and the best outcome was considered the final result, as shown in Table 4. The results of this study indicate that DL + ML were more effective than other features considered in this study for classification. Using the KNN classifier combined with DL + ML features, this work provided the highest classification accuracy of 99.33% for the FLAIR-modality MRI slice. Table 5 illustrates the results of implementing a similar procedure on the T2-modality MRI slice from TCIA. The proposed DL + ML technique achieved an accuracy of 99.67% with SVM. These results confirm that the proposed scheme is more effective at detecting the BT when using FLAIR-/T2-modulation MRI.

**Figure 8:**
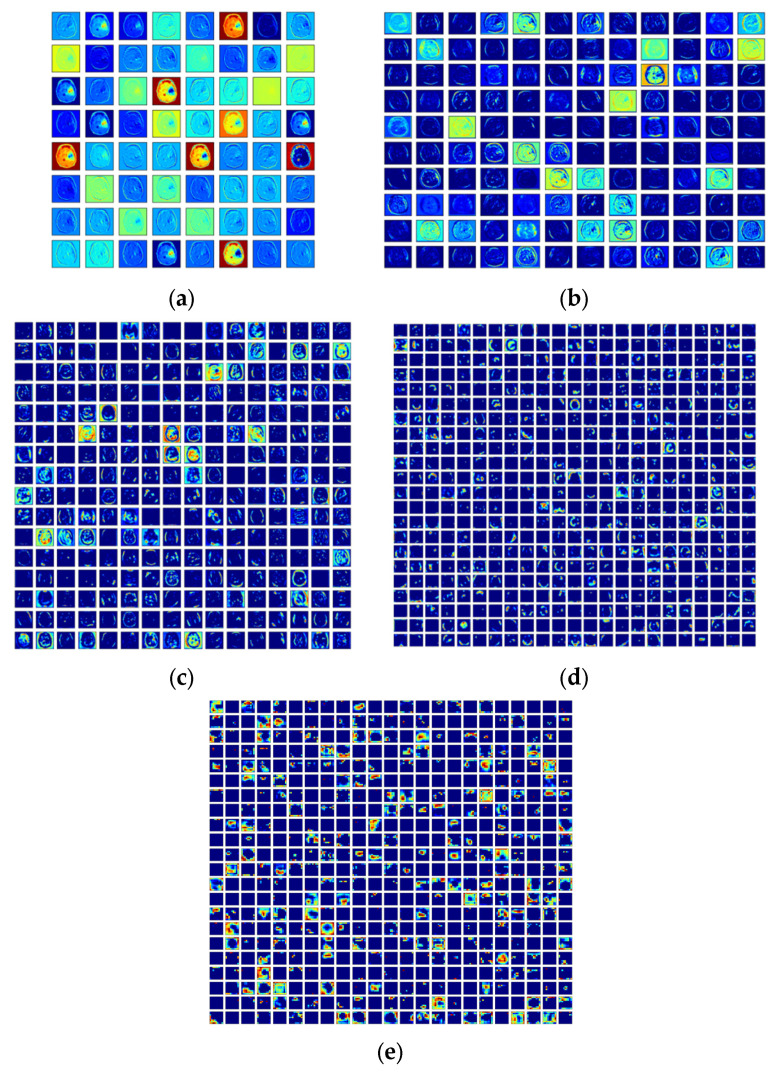
Various convolutional outcomes achieved during the DL + ML-based classification. (**a**) Convolution 1, (**b**) convolution 2, (**c**) convolution 3, (**d**) convolution 4, (**e**) convolution 5.

**Figure 9:**
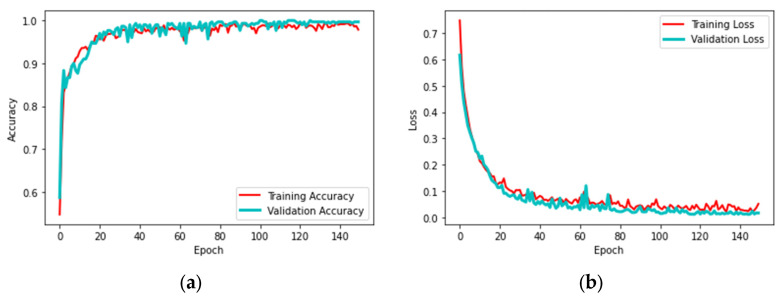
Training and validation outcome achieved with DL + ML classification. (**a**) Accuracy, (**b**) loss.

**Figure 10:**
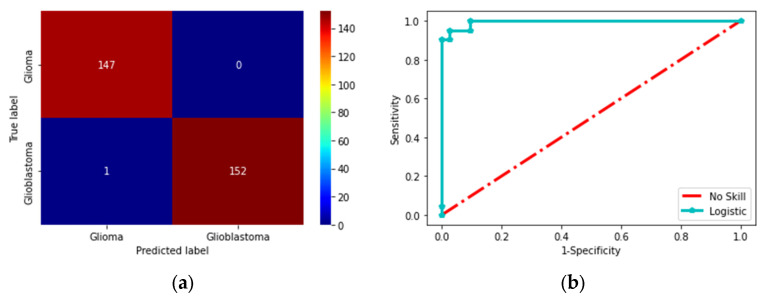
Confusion-matrix and RoC achieved with DL + ML classification. (**a**) CM, (**b**) RoC.

This scheme was initially tested using the BRATS database, and then the same scheme was considered to analyze TCIA. Hence, this work provided a better detection result with TCIA compared to BRATS. The experimental result achieved in this research for DL + ML-based detection of BT for the TCIA dataset is presented in Figure 8. Figure 8a–e show the results of convolutional layers 1 to 5. Figure 8 presents the final result after training the system on the database. This confirms that the training and validation results in Figure 9a,b are better. The overall performance of the proposed scheme was verified using the confusion matrix (CM) and receiver-operating-characteristic (RoC) curve, as presented in Figure 10a,b. This confirms that the proposed scheme worked well for the considered task. Furthermore, the proposed works show that the CM achieved was superior, with better TP, FN, TN, and FP values. Moreover, the RoC achieved in this work showed an area under curve (AUC) of 0.972, confirming the proposed technique’s ability.

**Figure 11:**
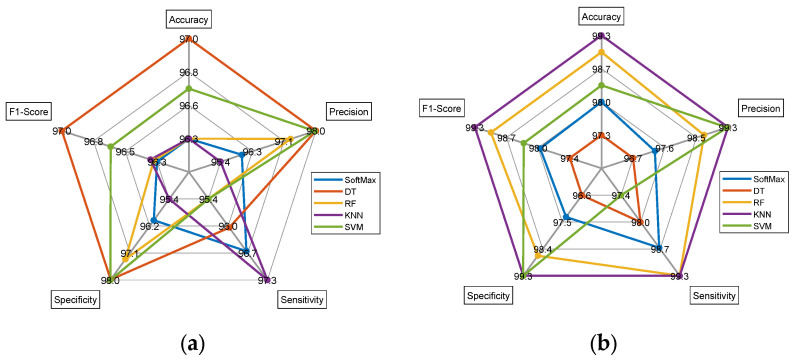

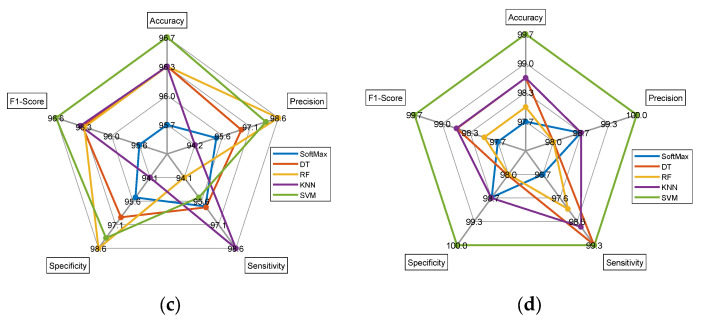
Spider plot achieved with the classification results of FLAIR- and T2-modality MRI. (**a**) Performance measure for FLAIR-modality dual deep features, (**b**) performance measure for FLAIR-modality DL + ML features, (**c**) Performance measure for T2-modality dual deep features, (**d**) Performance measure for T2 modality DL + ML features.

The commonly used spider plot was constructed using values from Table 4 and Table 5 to demonstrate the attained metrics using a graphical representation. Figure 11a,b present the results of the dual deep and DL + ML features for FLAIR-modality MRI. Figure 11c,d demonstrate the outcome achieved for the T2-modality MRI. These results confirm that the proposed technique helped to achieve a better outcome for the chosen MRI database and helped to achieve a better outcome for both of these modalities.

The investigational outcome of this study confirms that this scheme provided a better result and worked well on multi-modality MRI. The results achieved using (i) individual, (ii) dual deep, and (iii) fused DL + ML features provided a detection accuracy of >91% for LLG/HGGs and LGG/GBMs. Compared to the accuracies available in earlier works (Table 1), the proposed scheme provided a better result.

To confirm the merit of the work, the proposed scheme was also tested and validate on the LGG and GBM clinical databases considered in [48,49]. The experimental outcome confirms that this scheme helped obtain a detection accuracy of 98.92% with dual deep features and 98.95% with DL + ML, which is better than that of the earlier work by Rajinikanth et al. [5]. This result confirms that the executed procedure can work well on clinical MRI slices collected from patients.

The limitation of the proposed scheme is that it implements WS segmentation to extract the tumor section. In the future, a DL-segmentation technique can be considered to mine the tumor region to obtain the necessary ML features. Further, the merit of this architecture can be tested and verified using pretrained lightweight DL schemes found in the literature.

## 5. Conclusions

This research aims to develop and implement a procedure for analyzing tumors in FLAIR-/T2-MRI slices. For the assessment, axial-plane MRI slices were used. In addition, this study implemented DL and DL + ML-based procedures to improve the detection accuracy of MRI slices with or without skull sections. In the proposed experimental work, the VGG16 scheme was implemented to detect LGG/HGGs and LGG/GBMs from the considered databases. This study’s experimental results confirm that the proposed scheme worked well on the selected benchmark images, and its effectiveness can be verified with more clinical images in the future. Furthermore, when dual deep and DL + ML features were considered, this scheme achieved a higher BT-detection accuracy (accuracy > 99%). The results show that this approach worked well on MRI slices without the skull, and in the future, a skull-stripping procedure can be included in the preprocessing section to remove the skull. The experimental outcome of this research confirms that the proposed technique provides a better result and can be applied to the accurate analysis of patient data in the future.

## Figures and Tables

**Table 1:** Summary of MRI-based BT detection implemented with the DL scheme.

References	Procedure Employed	Accuracy (%)
Kalaiselvi et al. [24]	Convolutional-neural-network (CNN)-supported examination of BT in the BRATS database.	99.00
Raja [25]	The implementation of a deep autoencoder along Bayesian fuzzy-clustering segmentation is discussed to detect BT in the BRATS database.	98.50
Amin et al. [26]	Detection of BT in BRATS is presented using stacked autoencoders.	98.00
Özyurt and Avcı [27]	This work implements fuzzy c-means-based superpixel detection and CNN with an extreme-learning machine to detect BT in a TCIA dataset.	98.33
Anilkumar and Kumar [28]	BT in the BRATS database is assessed using transfer learning and KNN classification.	97.28
Sharif et al. [29]	Deep-transfer-learning-supported segmentation and classification are performed using MRI slices from BRATS.	92.00
Han et al. [30]	Data augmentation and classification of MRI slices from BRATS are performed using the CNN approach.	91.00
Amin et al. [31]	Transfer learning with score-level fusion to detect BT in MRI slices from the BRATS database.	99.00
Siar and Teshnehlab [32]	Integrated DL and ML approaches are presented to detect BT in MRI slices from the BRATS database.	87.00
Krishnammal and Raja [33]	Employment of CNN-based classification and BT-severity detection is performed using BRATS.	98.00
Ezhilarasi and Varalakshmi [34]	R-CNN scheme-based detection of BT from the BRATS database is discussed.	97.50
Antony et al. [35]	Automatic detection of BT using BRATS and CNN is presented.	97.00
Pandian and Balasubramanian [36]	Implementation of content-based image retrieval is discussed using TCIA brain-MRI slices.	88.00
Gudigar et al. [37]	Cascaded autoencoder-based feature fusion and binary classification are implemented to detect BT in T2-modality MRI slices from TCIA.	96.70
Demir et al. [38]	A novel CNN scheme is implemented to examine multi-modality brain MRIs from BRATS.	99.29
Qureshi et al. [39]	Deep-learning radiomic-feature-extraction-based automatic detection of brain MRI is proposed for the BRATS database.	96.84
Shelatkar et al. [40]	Automatic examination of a tumor in an MRI slice with a lightweight deep-learning scheme.	-

**Table 2:** The MRI slices considered in this work to verify the performance.

Image	Dimensions	Total	Training	Validation	Testing
Class1	224 × 224 × 3	1500	1200	150	150
Class2	224 × 224 × 3	1500	1200	150	150

**Table 3:** Performance metrics computed during DL-feature-based classification with SoftMax.

BT	Scheme	TP	FN	TN	FP	AC	PR	SE	SP	F1S
LGG/ HGG	VGG16	139	10	138	13	92.3333	91.4474	93.2886	91.3907	92.3588
	DenseNet101	135	14	140	11	91.6667	92.4658	90.6040	92.7152	91.5254
	ResNet101	136	13	134	17	90.0000	88.8889	91.2752	88.7417	90.0662
	VGG19	133	19	136	12	89.6667	91.7241	87.5000	91.8919	89.5623
	ResNet50	133	17	135	15	89.3333	89.8649	88.6667	90.0000	89.2617
LGG/ GBM	VGG16	138	14	138	10	92.0000	93.2432	90.7895	93.2432	92.0000
	DenseNet101	136	13	138	13	91.3333	91.2752	91.2752	91.3907	91.2752
	VGG19	134	19	136	11	90.0000	92.4138	87.5817	92.5170	89.9329
	ResNet101	131	18	137	14	89.3333	90.3448	87.9195	90.7285	89.1156
	ResNet50	132	17	135	16	89.0000	89.1892	88.5906	89.4040	88.8889

**Table 4:** Metrics achieved with dual deep and fused features for FLAIR-modality MRI.

Features	Classifiers	TP	FN	TN	FP	AC	PR	SE	SP	F1S
Dual Deep	SoftMax	143	5	146	6	96.3333	95.9732	96.6216	96.0526	96.2963
	DT	146	6	145	3	97.0000	97.9866	96.0526	97.9730	97.0100
	RF	144	7	145	4	96.3333	97.2973	95.3642	97.3154	96.3211
	KNN	145	4	144	7	96.3333	95.3947	97.3154	95.3642	96.3455
	SVM	144	7	146	3	96.6667	97.9592	95.3642	97.9866	96.6443
DL + ML	SoftMax	146	2	148	4	98.0000	97.3333	98.6486	97.3684	97.9866
	DT	148	3	144	5	97.3333	96.7320	98.0132	96.6443	97.3684
	RF	150	1	147	2	99.0000	98.6842	99.3377	98.6577	99.0099
	KNN	151	1	147	1	99.3333	99.3421	99.3421	99.3243	99.3421
	SVM	147	4	148	1	98.3333	99.3243	97.3510	99.3289	98.3278

**Table 5:** Metrics achieved with dual deep and fused features for T2-modality MRI.

Features	Classifiers	TP	FN	TN	FP	AC	PR	SE	SP	F1S
Dual-Deep	SoftMax	142	6	145	7	95.6667	95.3020	95.9459	95.3947	95.6229
	DT	144	6	145	5	96.3333	96.6443	96.0000	96.6667	96.3211
	RF	143	9	146	2	96.3333	98.6207	94.0789	98.6486	96.2963
	KNN	145	2	144	9	96.3333	94.1558	98.6395	94.1176	96.3455
	SVM	144	7	146	3	96.6667	97.9592	95.3642	97.9866	96.6443
DL + ML	SoftMax	146	5	147	2	97.6667	98.6486	96.6887	98.6577	97.6589
	DT	146	1	150	3	98.6667	97.9866	99.3197	98.0392	98.6486
	RF	146	3	148	3	98.0000	97.9866	97.9866	98.0132	97.9866
	KNN	147	2	149	2	98.6667	98.6577	98.6577	98.6755	98.6577
	SVM	152	1	147	0	99.6667	100	99.3464	100	99.6721

## Data Availability

The databases considered in this research work can be accessed from; (i) https://www.smir.ch/BRATS/Start2015 (accessed on 11 December 2022), (ii) https://wiki.cancerimagingarchive.net/pages/viewpage.action?pageId=5309188 (accessed on 11 December 2022) (iii) https://wiki.cancerimagingarchive.net/pages/viewpage.action?pageId=1966258 (accessed on 11 December 2022).
